# 2-(3,4-Dimethyl­anilino)acetohydrazide

**DOI:** 10.1107/S1600536809038963

**Published:** 2009-09-30

**Authors:** Muhammad Salim, Zaid Mahmood, M. Nawaz Tahir, Saeed Ahmad, Muhammad Yaseen

**Affiliations:** a Institute of Chemistry, University of the Punjab, Lahore, Pakistan; b Department of Physics, University of Sargodha, Sargodha, Pakistan; c Department of Chemistry, Gomal University, Dera Ismail Khan, Pakistan

## Abstract

The title compound, C_10_H_15_N_3_O, crystallizes in an infinite two-dimensional polymeric network due to inter­molecular N—H⋯O hydrogen bonding. Intra­molecular N—H⋯N and inter­molecular C—H⋯N inter­actions are also present. The 3,4-dimethyl­phenyl unit is disordered over two sites with an occupancy ratio of 0.677 (5):0.323 (5). The dihedral angle between the benzene rings of the disordered components is 2.6 (6)°.

## Related literature

For the structure of phenyl­glycine hydrazide, see: Gudasi *et al.* (2007 ▶). For the biological and medicinal activity of hydrazide compounds, see: Hall *et al.* (1993 ▶); Waisser *et al.* (1990 ▶).
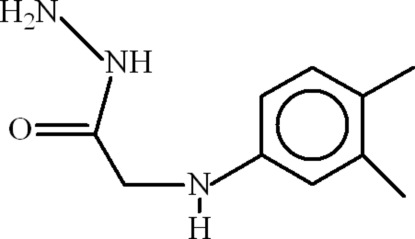


## Experimental

### 

#### Crystal data

C_10_H_15_N_3_O
*M* *_r_* = 193.25
Triclinic, 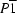

*a* = 5.1956 (6) Å
*b* = 6.0869 (7) Å
*c* = 16.3477 (19) Å
α = 80.657 (6)°
β = 86.733 (5)°
γ = 84.040 (6)°
*V* = 506.96 (10) Å^3^
*Z* = 2
Mo *K*α radiation
μ = 0.09 mm^−1^
*T* = 296 K
0.25 × 0.12 × 0.10 mm

#### Data collection

Bruker Kappa APEXII CCD diffractometer
Absorption correction: multi-scan (*SADABS*; Bruker, 2005 ▶) *T*_min_ = 0.989, *T*_max_ = 0.991
8717 measured reflections
2182 independent reflections
1299 reflections with *I* > 2σ(*I*)
*R*_int_ = 0.034

#### Refinement

*R*[*F* ^2^ > 2σ(*F* ^2^)] = 0.053
*wR*(*F* ^2^) = 0.157
*S* = 1.03
2182 reflections
180 parameters
1 restraint
H atoms treated by a mixture of independent and constrained refinement
Δρ_max_ = 0.22 e Å^−3^
Δρ_min_ = −0.31 e Å^−3^

### 

Data collection: *APEX2* (Bruker, 2007 ▶); cell refinement: *SAINT* (Bruker, 2007 ▶); data reduction: *SAINT*; program(s) used to solve structure: *SHELXS97* (Sheldrick, 2008 ▶); program(s) used to refine structure: *SHELXL97* (Sheldrick, 2008 ▶); molecular graphics: *ORTEP-3 for Windows* (Farrugia, 1997 ▶) and *PLATON* (Spek, 2009 ▶); software used to prepare material for publication: *WinGX* (Farrugia, 1999 ▶) and *PLATON*.

## Supplementary Material

Crystal structure: contains datablocks global, I. DOI: 10.1107/S1600536809038963/si2205sup1.cif

Structure factors: contains datablocks I. DOI: 10.1107/S1600536809038963/si2205Isup2.hkl

Additional supplementary materials:  crystallographic information; 3D view; checkCIF report

## Figures and Tables

**Table 1:** Hydrogen-bond geometry (Å, °)

*D*—H⋯*A*	*D*—H	H⋯*A*	*D*⋯*A*	*D*—H⋯*A*
N1—H1⋯O1^i^	0.8600	2.5200	3.223 (2)	140.00
N2—H2⋯N1	0.8600	2.2500	2.672 (3)	110.00
N2—H2⋯N3^ii^	0.8600	2.4700	3.139 (3)	136.00
N3—H3*A*⋯O1	0.91 (3)	2.39 (2)	2.779 (3)	105.6 (17)
N3—H3*A*⋯O1^iii^	0.91 (3)	2.42 (2)	3.223 (2)	147 (2)
N3—H3*B*⋯O1^iv^	0.93 (3)	2.32 (3)	3.156 (3)	149 (2)
C9—H9*B*⋯N3^v^	0.9700	2.5800	3.484 (3)	155.00

Symmetry codes: (i) 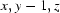; (ii) 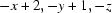; (iii) 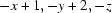; (iv) 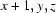; (v) 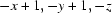.

**Fig. 1.**
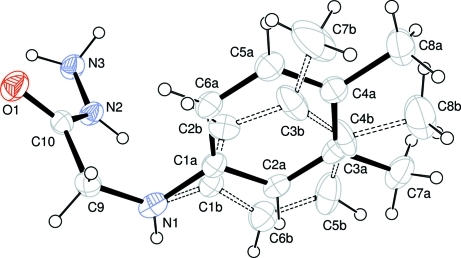
View of the title compound with the atom numbering scheme. The thermal ellipsoids are drawn at the 30% probability level. H-atoms are shown by small circles of arbitrary radii. The dotted lines represent the group of lower occupancy factor.

**Fig. 2.**
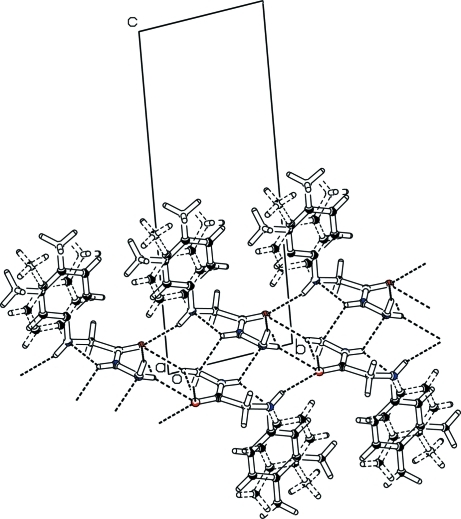
The projectional view (PLATON; Spek, 2009) along the a axis which shows that the molecules are stabilized in form of a two dimensional polymeric network.

## supplementary crystallographic information

### Comment

The hydrazides and their analogues are known to have different biological activities such as tuberculostatic activity, antifungal and monoamine oxidase inhibitory activity (Waisser *et al.*, 1990; Hall *et al.*, 1993). The title compound (I, Fig. 1) has been prepared as an intermediate for further derivatization with various substituted pyridine aldehydes.

The crystal structure of (II) Phenylglycine hydrazide (Gudasi *et al.*, 2007) and the title compound (I) differ due to substitution of the methyl moieties. In the title compound, the 3,4-dimethylphenyl group is disordered over two possible sites with an occupancy ratio 0.677 (5):0.323 (5). The dihedral angle between the benzene rings A (C1A—C6A) and B (C1B—C6B) of the disordered moiety is 2.6 (6)°. The group C (N1/N2/N3/C10/O1) is almost planar with maximum r.m.s. deviation of 0.0457 Å from its mean square plane, and C9 is at a distance of -0.2594 (26) Å. The dihedral angle between A/C and B/C is 89.46 (10) and 87.80 (23)°, respectively. The title compound is stabilized in the form of an infinite two dimensional polymeric network due to intra as well as inter-molecular N—H···O hydrogen bondings (Table 1, Fig. 2).

### Experimental

In a first step ethylchloroacetate (2.3 g, 0.0187 mol), the 3,4-dimethylaniline (2.266 g, 0.0187 mol) and triethylamine (1.89 g, 0.0187 mol) were refluxed in 60 ml of THF. The reaction was monitored by TLC and solvent was removed under reduced pressure. The solid residue obtained was washed with water to get reddish precipitate of ethyl [(3,4-dimethylphenyl)amino]acetate.

In a second step ethyl[(3,4-dimethylphenyl)amino]acetate (3.41 g, 0.0164 mol) and about three folds of hydrazine hydrate (2.46 g, 0.0492 mol) were refluxed in 20 ml of ethyl alcohol. On evaporation of solvent at room temperature yellow needles of the title compound (I) were obtained.

### Refinement

The coordinates of H-atoms of NH_2_ group were refined. H-atoms were positioned geometrically, with N—H = 0.86 Å for NH group, C—H = 0.93, 0.96 and 0.97 Å for aryl, methyl and methylene H, respectively and constrained to ride on their parent atoms, with *U*_iso_(H) = *xU*_eq_(C, N), where *x* = 1.5 for methyl and 1.2 for all other H atoms.

The benzene rings of the disordered group were fitted using AFIX 66.

### Crystal data

**Table:**

C_10_H_15_N_3_O	*Z* = 2
*M**_r_* = 193.25	*F*(000) = 208
Triclinic, *P*1	*D*_x_ = 1.266 Mg m^−^^3^
Hall symbol: -P 1	Mo *K*α radiation, λ = 0.71073 Å
*a* = 5.1956 (6) Å	Cell parameters from 2187 reflections
*b* = 6.0869 (7) Å	θ = 2.5–27.1°
*c* = 16.3477 (19) Å	µ = 0.09 mm^−^^1^
α = 80.657 (6)°	*T* = 296 K
β = 86.733 (5)°	Needle, yellow
γ = 84.040 (6)°	0.25 × 0.12 × 0.10 mm
*V* = 506.96 (10) Å^3^	

### Data collection

**Table:**

Bruker Kappa APEXII CCD diffractometer	2182 independent reflections
Radiation source: fine-focus sealed tube	1299 reflections with *I* > 2σ(*I*)
graphite	*R*_int_ = 0.034
Detector resolution: 7.60 pixels mm^-1^	θ_max_ = 27.1°, θ_min_ = 2.5°
ω scans	*h* = −6→5
Absorption correction: multi-scan (*SADABS*; Bruker, 2005)	*k* = −7→7
*T*_min_ = 0.989, *T*_max_ = 0.991	*l* = −20→19
8717 measured reflections	

### Refinement

**Table:**

Refinement on *F*^2^	Primary atom site location: structure-invariant direct methods
Least-squares matrix: full	Secondary atom site location: difference Fourier map
*R*[*F*^2^ > 2σ(*F*^2^)] = 0.053	Hydrogen site location: inferred from neighbouring sites
*wR*(*F*^2^) = 0.157	H atoms treated by a mixture of independent and constrained refinement
*S* = 1.03	*w* = 1/[σ^2^(*F*_o_^2^) + (0.0718*P*)^2^ + 0.1107*P*] where *P* = (*F*_o_^2^ + 2*F*_c_^2^)/3
2182 reflections	(Δ/σ)_max_ < 0.001
180 parameters	Δρ_max_ = 0.22 e Å^−^^3^
1 restraint	Δρ_min_ = −0.30 e Å^−^^3^

### Special details

**Table:**

Geometry. Bond distances, angles *etc*. have been calculated using the rounded fractional coordinates. All su's are estimated from the variances of the (full) variance-covariance matrix. The cell e.s.d.'s are taken into account in the estimation of distances, angles and torsion angles.
Refinement. Refinement of *F*^2^ against ALL reflections. The weighted *R*-factor *wR* and goodness of fit *S* are based on *F*^2^, conventional *R*-factors *R* are based on *F*, with *F* set to zero for negative *F*^2^. The threshold expression of *F*^2^ > σ(*F*^2^) is used only for calculating *R*-factors(gt) *etc*. and is not relevant to the choice of reflections for refinement. *R*-factors based on *F*^2^ are statistically about twice as large as those based on *F*, and *R*- factors based on ALL data will be even larger.

### Fractional atomic coordinates and isotropic or equivalent isotropic displacement parameters (Å^2^)

**Table:**

	*x*	*y*	*z*	*U*_iso_*/*U*_eq_	Occ. (<1)
O1	0.3981 (3)	0.8061 (2)	0.09951 (10)	0.0591 (6)	
N1	0.6054 (3)	0.2257 (3)	0.16440 (12)	0.0503 (6)	
N2	0.7511 (3)	0.5889 (3)	0.06510 (10)	0.0427 (6)	
N3	0.8768 (4)	0.7634 (3)	0.01577 (12)	0.0488 (6)	
C1A	0.7662 (6)	0.2289 (5)	0.2378 (2)	0.0393 (10)	0.677 (5)
C2A	0.9473 (5)	0.0477 (4)	0.2606 (2)	0.0438 (13)	0.677 (5)
C3A	1.1022 (5)	0.0453 (4)	0.3274 (2)	0.0490 (17)	0.677 (5)
C4A	1.0758 (5)	0.2241 (5)	0.37135 (17)	0.0477 (14)	0.677 (5)
C5A	0.8947 (5)	0.4053 (5)	0.34854 (18)	0.0533 (14)	0.677 (5)
C6A	0.7398 (6)	0.4077 (5)	0.2818 (2)	0.0467 (12)	0.677 (5)
C7A	1.3044 (7)	−0.1488 (7)	0.3490 (3)	0.0738 (16)	0.677 (5)
C8A	1.2393 (8)	0.2247 (8)	0.4457 (2)	0.0712 (14)	0.677 (5)
C9	0.4124 (4)	0.4092 (3)	0.14708 (14)	0.0462 (7)	
C10	0.5202 (4)	0.6210 (3)	0.10230 (13)	0.0404 (6)	
C1B	0.7655 (13)	0.1771 (12)	0.2182 (4)	0.0393 (10)	0.323 (5)
C2B	0.7810 (13)	0.3188 (12)	0.2760 (4)	0.044 (3)	0.323 (5)
C3B	0.9597 (14)	0.2633 (16)	0.3379 (4)	0.070 (4)	0.323 (5)
C4B	1.1229 (12)	0.0662 (18)	0.3420 (4)	0.070 (6)	0.323 (5)
C5B	1.1073 (11)	−0.0755 (13)	0.2842 (5)	0.082 (4)	0.323 (5)
C6B	0.9286 (13)	−0.0200 (11)	0.2222 (5)	0.061 (3)	0.323 (5)
C7B	0.976 (3)	0.398 (2)	0.4034 (7)	0.128 (7)	0.323 (5)
C8B	1.327 (2)	−0.016 (2)	0.4075 (7)	0.107 (5)	0.323 (5)
H9B	0.28341	0.36754	0.11331	0.0555*	
H71	1.47378	−0.09809	0.33824	0.1105*	0.677 (5)
H72	1.28469	−0.26206	0.31590	0.1105*	0.677 (5)
H73	1.28350	−0.20965	0.40670	0.1105*	0.677 (5)
H81	1.22173	0.09055	0.48469	0.1067*	0.677 (5)
H82	1.18148	0.35253	0.47170	0.1067*	0.677 (5)
H83	1.41764	0.23127	0.42745	0.1067*	0.677 (5)
H1	0.62840	0.12010	0.13484	0.0604*	
H2	0.82856	0.45577	0.07117	0.0512*	
H2A	0.96495	−0.07194	0.23116	0.0527*	0.677 (5)
H3A	0.750 (5)	0.876 (4)	0.0011 (14)	0.0586*	
H3B	0.987 (5)	0.812 (4)	0.0509 (14)	0.0586*	
H5A	0.87706	0.52492	0.37797	0.0638*	0.677 (5)
H6A	0.61862	0.52888	0.26650	0.0560*	0.677 (5)
H9A	0.32655	0.43974	0.19886	0.0555*	
H2B	0.67185	0.45070	0.27324	0.0524*	0.323 (5)
H5B	1.21645	−0.20739	0.28692	0.0981*	0.323 (5)
H6B	0.91819	−0.11483	0.18355	0.0722*	0.323 (5)
H74	0.94121	0.30939	0.45637	0.1919*	0.323 (5)
H75	0.85030	0.52584	0.39513	0.1919*	0.323 (5)
H76	1.14620	0.44622	0.40183	0.1919*	0.323 (5)
H84	1.24727	−0.09990	0.45535	0.1600*	0.323 (5)
H85	1.39623	0.11061	0.42324	0.1600*	0.323 (5)
H86	1.46349	−0.10908	0.38474	0.1600*	0.323 (5)

### Atomic displacement parameters (Å^2^)

**Table:**

	*U*^11^	*U*^22^	*U*^33^	*U*^12^	*U*^13^	*U*^23^
O1	0.0463 (8)	0.0453 (9)	0.0813 (12)	0.0028 (7)	0.0029 (8)	−0.0035 (8)
N1	0.0550 (11)	0.0357 (9)	0.0591 (12)	−0.0053 (8)	0.0063 (9)	−0.0063 (8)
N2	0.0367 (9)	0.0339 (9)	0.0541 (11)	−0.0060 (7)	0.0040 (8)	0.0024 (8)
N3	0.0415 (10)	0.0403 (10)	0.0604 (13)	−0.0108 (8)	0.0008 (9)	0.0078 (9)
C1A	0.0391 (12)	0.038 (2)	0.038 (2)	−0.0060 (14)	0.0067 (14)	0.0007 (17)
C2A	0.0480 (19)	0.0329 (18)	0.049 (3)	−0.0067 (15)	0.0062 (18)	−0.0031 (16)
C3A	0.045 (3)	0.035 (3)	0.061 (3)	−0.002 (2)	0.013 (2)	0.004 (2)
C4A	0.048 (2)	0.048 (2)	0.046 (3)	−0.0130 (18)	0.0021 (17)	−0.0001 (19)
C5A	0.056 (2)	0.048 (2)	0.055 (3)	−0.0001 (17)	0.0026 (19)	−0.0103 (19)
C6A	0.048 (2)	0.039 (2)	0.052 (2)	0.0053 (17)	0.0013 (18)	−0.0115 (18)
C7A	0.052 (2)	0.054 (2)	0.104 (4)	0.0030 (18)	−0.003 (2)	0.016 (2)
C8A	0.065 (2)	0.087 (3)	0.058 (2)	−0.013 (2)	−0.0144 (19)	0.007 (2)
C9	0.0370 (10)	0.0506 (12)	0.0503 (13)	−0.0124 (9)	−0.0015 (9)	−0.0006 (10)
C10	0.0338 (9)	0.0408 (11)	0.0461 (12)	−0.0045 (9)	−0.0039 (9)	−0.0042 (9)
C1B	0.0391 (12)	0.038 (2)	0.038 (2)	−0.0060 (14)	0.0067 (14)	0.0007 (17)
C2B	0.046 (4)	0.051 (6)	0.038 (5)	−0.007 (4)	0.013 (4)	−0.022 (4)
C3B	0.055 (6)	0.114 (10)	0.043 (6)	−0.044 (6)	−0.007 (4)	0.008 (6)
C4B	0.042 (7)	0.116 (15)	0.047 (6)	−0.040 (8)	−0.017 (5)	0.026 (8)
C5B	0.048 (5)	0.091 (7)	0.088 (7)	0.019 (5)	0.002 (5)	0.023 (6)
C6B	0.069 (5)	0.060 (5)	0.051 (6)	−0.003 (4)	0.000 (4)	−0.007 (4)
C7B	0.178 (13)	0.165 (13)	0.055 (7)	−0.084 (11)	−0.038 (7)	−0.006 (7)
C8B	0.080 (7)	0.141 (12)	0.091 (8)	−0.030 (7)	−0.027 (6)	0.025 (8)

### Geometric parameters (Å, °)

**Table:**

O1—C10	1.230 (2)	C5A—C6A	1.390 (4)
N1—C1A	1.505 (4)	C5B—C6B	1.391 (10)
N1—C9	1.425 (3)	C9—C10	1.519 (3)
N1—C1B	1.225 (7)	C2A—H2A	0.9300
N2—N3	1.418 (3)	C2B—H2B	0.9300
N2—C10	1.324 (3)	C5A—H5A	0.9300
N1—H1	0.8600	C5B—H5B	0.9300
N2—H2	0.8600	C6A—H6A	0.9300
N3—H3A	0.91 (3)	C6B—H6B	0.9300
N3—H3B	0.93 (3)	C7A—H71	0.9600
C1A—C2A	1.390 (4)	C7A—H72	0.9600
C1A—C6A	1.390 (4)	C7A—H73	0.9600
C1B—C2B	1.390 (10)	C7B—H74	0.9600
C1B—C6B	1.390 (10)	C7B—H75	0.9600
C2A—C3A	1.390 (4)	C7B—H76	0.9600
C2B—C3B	1.390 (10)	C8A—H81	0.9600
C3A—C7A	1.508 (5)	C8A—H82	0.9600
C3A—C4A	1.390 (4)	C8A—H83	0.9600
C3B—C4B	1.390 (13)	C8B—H84	0.9600
C3B—C7B	1.461 (14)	C8B—H86	0.9600
C4A—C8A	1.523 (5)	C8B—H85	0.9600
C4A—C5A	1.390 (4)	C9—H9B	0.9700
C4B—C5B	1.390 (12)	C9—H9A	0.9700
C4B—C8B	1.538 (13)		
			
O1···N1^i^	3.223 (2)	H2···N1	2.2500
O1···N3^ii^	3.156 (3)	H2···H9B^vii^	2.4800
O1···N3	2.779 (3)	H2···C1B	2.7300
O1···C6B^iii^	3.269 (7)	H2···H1	2.4400
O1···N3^iv^	3.223 (2)	H2···N3^vi^	2.4700
O1···H3A	2.39 (2)	H2A···H72	2.3200
O1···H1^i^	2.5200	H2A···H1	2.4900
O1···H6B^iii^	2.8100	H2B···C10	2.9400
O1···H3B^ii^	2.32 (3)	H2B···C9	2.5900
O1···H3A^iv^	2.42 (2)	H2B···H75	2.3700
N1···O1^v^	3.223 (2)	H2B···H9A	2.2400
N1···N2	2.672 (3)	H3A···O1^iv^	2.42 (2)
N2···C1B	3.240 (7)	H3A···O1	2.39 (2)
N2···N1	2.672 (3)	H3A···H9B^ix^	2.5900
N2···C1A	3.280 (4)	H3A···H3B^viii^	2.47 (4)
N2···N3^vi^	3.139 (3)	H3B···C10^vii^	3.00 (3)
N3···O1^vii^	3.156 (3)	H3B···O1^vii^	2.32 (3)
N3···O1^iv^	3.223 (2)	H3B···H3A^viii^	2.47 (4)
N3···O1	2.779 (3)	H3B···H6B^i^	2.2800
N3···N3^viii^	3.226 (3)	H3B···N3^viii^	2.78 (2)
N3···N2^vi^	3.139 (3)	H5A···H82	2.3200
N1···H2	2.2500	H5A···C7A^i^	3.1000
N2···H9B^ix^	2.9000	H5B···H86	2.2900
N3···H2^vi^	2.4700	H6A···C9	2.5200
N3···H3B^viii^	2.78 (2)	H6A···C10	2.7200
N3···H9B^ix^	2.5800	H6A···H72^iii^	2.2400
C1A···N2	3.280 (4)	H6A···H9A	2.0900
C1B···N2	3.240 (7)	H6A···C7A^iii^	2.8800
C2B···C10	3.392 (7)	H6B···O1^x^	2.8100
C5B···C7B^v^	3.579 (15)	H6B···H1	2.0700
C6A···C10	3.230 (4)	H6B···H3B^v^	2.2800
C6B···O1^x^	3.269 (7)	H9A···C6A	2.5800
C7B···C5B^i^	3.579 (15)	H9A···C2B	2.7200
C10···C2B	3.392 (7)	H9A···C3B^ii^	3.0200
C10···C6A	3.230 (4)	H9A···H6A	2.0900
C1A···H2	2.8600	H9A···H2B	2.2400
C1A···H71^ii^	2.8700	H9B···N2^ix^	2.9000
C1B···H2	2.7300	H9B···N3^ix^	2.5800
C2A···H71^ii^	2.8600	H9B···H2^ii^	2.4800
C2B···H9A	2.7200	H9B···H3A^ix^	2.5900
C3B···H9A^vii^	3.0200	H71···C1A^vii^	2.8700
C5A···H72^i^	2.9800	H71···C2A^vii^	2.8600
C5A···H83^ii^	2.9400	H71···C8A	2.9700
C6A···H72^iii^	3.0300	H72···H6A^x^	2.2400
C6A···H9A	2.5800	H72···C6A^x^	3.0300
C6A···H83^ii^	2.9500	H72···H2A	2.3200
C7A···H5A^v^	3.1000	H72···C5A^v^	2.9800
C7A···H6A^x^	2.8800	H73···C8A	2.8000
C7A···H81	2.8400	H73···H81	2.3800
C7A···H83	2.9500	H74···C8B	2.8400
C7B···H85	2.6500	H74···H84^xi^	2.0400
C7B···H84^xi^	2.9600	H74···C8B^xi^	2.9900
C8A···H71	2.9700	H75···H2B	2.3700
C8A···H73	2.8000	H76···C8B	2.8600
C8B···H76	2.8600	H76···H85	2.2900
C8B···H74	2.8400	H81···H73	2.3800
C8B···H74^xi^	2.9900	H81···C7A	2.8400
C9···H2B	2.5900	H82···H5A	2.3200
C9···H6A	2.5200	H83···C6A^vii^	2.9500
C10···H3B^ii^	3.00 (3)	H83···C7A	2.9500
C10···H6A	2.7200	H83···C5A^vii^	2.9400
C10···H2B	2.9400	H84···C7B^xi^	2.9600
H1···H6B	2.0700	H84···H74^xi^	2.0400
H1···H2	2.4400	H85···C7B	2.6500
H1···H2A	2.4900	H85···H76	2.2900
H1···O1^v^	2.5200	H86···H5B	2.2900
H2···C1A	2.8600		
			
C1A—N1—C9	115.60 (19)	C3A—C2A—H2A	120.00
C1B—N1—C9	132.4 (4)	C3B—C2B—H2B	120.00
N3—N2—C10	123.20 (17)	C1B—C2B—H2B	120.00
C1B—N1—H1	105.00	C6A—C5A—H5A	120.00
C9—N1—H1	122.00	C4A—C5A—H5A	120.00
C1A—N1—H1	122.00	C6B—C5B—H5B	120.00
N3—N2—H2	118.00	C4B—C5B—H5B	120.00
C10—N2—H2	118.00	C5A—C6A—H6A	120.00
N2—N3—H3A	105.9 (16)	C1A—C6A—H6A	120.00
N2—N3—H3B	105.9 (14)	C5B—C6B—H6B	120.00
H3A—N3—H3B	108 (2)	C1B—C6B—H6B	120.00
C2A—C1A—C6A	120.0 (3)	C3A—C7A—H72	110.00
N1—C1A—C2A	118.2 (3)	C3A—C7A—H71	109.00
N1—C1A—C6A	121.8 (3)	H71—C7A—H72	110.00
C2B—C1B—C6B	120.1 (6)	H71—C7A—H73	109.00
N1—C1B—C6B	120.3 (6)	C3A—C7A—H73	109.00
N1—C1B—C2B	119.6 (6)	H72—C7A—H73	109.00
C1A—C2A—C3A	120.0 (3)	C3B—C7B—H74	109.00
C1B—C2B—C3B	120.0 (7)	C3B—C7B—H75	109.00
C2A—C3A—C7A	119.1 (3)	C3B—C7B—H76	110.00
C4A—C3A—C7A	120.9 (3)	H74—C7B—H75	109.00
C2A—C3A—C4A	120.0 (2)	H74—C7B—H76	110.00
C2B—C3B—C4B	120.0 (7)	H75—C7B—H76	110.00
C4B—C3B—C7B	117.1 (8)	C4A—C8A—H82	109.00
C2B—C3B—C7B	122.8 (9)	C4A—C8A—H83	109.00
C5A—C4A—C8A	118.7 (3)	C4A—C8A—H81	109.00
C3A—C4A—C8A	121.2 (3)	H81—C8A—H82	110.00
C3A—C4A—C5A	120.0 (3)	H81—C8A—H83	109.00
C3B—C4B—C5B	120.0 (6)	H82—C8A—H83	110.00
C5B—C4B—C8B	114.7 (8)	C4B—C8B—H84	109.00
C3B—C4B—C8B	125.3 (8)	C4B—C8B—H85	109.00
C4A—C5A—C6A	120.0 (3)	C4B—C8B—H86	109.00
C4B—C5B—C6B	120.0 (7)	H85—C8B—H86	110.00
C1A—C6A—C5A	120.0 (3)	H84—C8B—H85	109.00
C1B—C6B—C5B	119.9 (7)	H84—C8B—H86	110.00
N1—C9—C10	113.32 (17)	N1—C9—H9B	109.00
O1—C10—C9	122.10 (19)	H9A—C9—H9B	108.00
N2—C10—C9	114.70 (17)	C10—C9—H9A	109.00
O1—C10—N2	123.18 (19)	C10—C9—H9B	109.00
C1A—C2A—H2A	120.00	N1—C9—H9A	109.00
			
C9—N1—C1A—C2A	176.3 (2)	C1A—C2A—C3A—C4A	0.0 (4)
C9—N1—C1A—C6A	−4.5 (4)	C7A—C3A—C4A—C5A	177.6 (3)
C1A—N1—C9—C10	78.7 (2)	C2A—C3A—C4A—C8A	178.9 (3)
N3—N2—C10—O1	−2.4 (3)	C2A—C3A—C4A—C5A	0.0 (4)
N3—N2—C10—C9	175.88 (18)	C7A—C3A—C4A—C8A	−3.4 (5)
N1—C1A—C2A—C3A	179.2 (2)	C8A—C4A—C5A—C6A	−179.0 (3)
N1—C1A—C6A—C5A	−179.1 (3)	C3A—C4A—C5A—C6A	0.0 (4)
C2A—C1A—C6A—C5A	0.1 (5)	C4A—C5A—C6A—C1A	−0.1 (4)
C6A—C1A—C2A—C3A	0.0 (5)	N1—C9—C10—N2	19.1 (3)
C1A—C2A—C3A—C7A	−177.7 (3)	N1—C9—C10—O1	−162.6 (2)

Symmetry codes: (i) *x*, *y*+1, *z*; (ii) *x*−1, *y*, *z*; (iii) *x*−1, *y*+1, *z*; (iv) −*x*+1, −*y*+2, −*z*; (v) *x*, *y*−1, *z*; (vi) −*x*+2, −*y*+1, −*z*; (vii) *x*+1, *y*, *z*; (viii) −*x*+2, −*y*+2, −*z*; (ix) −*x*+1, −*y*+1, −*z*; (x) *x*+1, *y*−1, *z*; (xi) −*x*+2, −*y*, −*z*+1.

### Hydrogen-bond geometry (Å, °)

**Table:**

*D*—H···*A*	*D*—H	H···*A*	*D*···*A*	*D*—H···*A*
N1—H1···O1^v^	0.8600	2.5200	3.223 (2)	140.00
N2—H2···N1	0.8600	2.2500	2.672 (3)	110.00
N2—H2···N3^vi^	0.8600	2.4700	3.139 (3)	136.00
N3—H3A···O1	0.91 (3)	2.39 (2)	2.779 (3)	105.6 (17)
N3—H3A···O1^iv^	0.91 (3)	2.42 (2)	3.223 (2)	147 (2)
N3—H3B···O1^vii^	0.93 (3)	2.32 (3)	3.156 (3)	149 (2)
C9—H9B···N3^ix^	0.9700	2.5800	3.484 (3)	155.00

Symmetry codes: (v) *x*, *y*−1, *z*; (vi) −*x*+2, −*y*+1, −*z*; (iv) −*x*+1, −*y*+2, −*z*; (vii) *x*+1, *y*, *z*; (ix) −*x*+1, −*y*+1, −*z*.
